# A combination of cherry juice and cold water immersion does not enhance marathon recovery compared to either treatment in isolation: A randomized placebo-controlled trial

**DOI:** 10.3389/fspor.2022.957950

**Published:** 2022-08-19

**Authors:** Isabella Difranco, Emma Cockburn, Lygeri Dimitriou, Katherine Paice, Scott Sinclair, Tanwir Faki, Frank A. Hills, Marcela B. Gondek, Alyssa Wood, Laura J. Wilson

**Affiliations:** ^1^School of Biomedical, Nutritional and Sport Sciences, Newcastle University, Newcastle upon Tyne, United Kingdom; ^2^Department of Natural Sciences, Middlesex University, London, United Kingdom; ^3^London Sports Institute, Middlesex University, London, United Kingdom; ^4^Faculty of Dance, Trinity Laban Conservatoire of Music and Dance, London, United Kingdom

**Keywords:** inflammation, muscle function, endurance athletes, cryotherapy, phytochemicals

## Abstract

**Purpose:**

Cherry juice (CJ) and cold water immersion (CWI) are both effective recovery strategies following strenuous endurance exercise. However, athletes routinely combine recovery interventions and less is known about the impact of a combined CJ and CWI protocol. Therefore, this study investigated the effects of combining CWI and CJ (a “cocktail” (CT)) on inflammation and muscle damage following a marathon.

**Methods:**

A total 39 endurance trained males were randomly assigned to a placebo (PL), CWI, CJ, or CT group before completing a trail marathon run. Muscle damage (creatine kinase (CK)), muscle function (maximal voluntary isometric contraction (MVIC)), and inflammation (interleukin-6 (IL-6); C-reactive protein (CRP)) were measured at baseline, immediately after marathon (only IL-6), 24 h, and 48 h after marathon.

**Results:**

There were no statistically significant differences between groups and no group × time interaction effects for any of the dependent variables. Confidence intervals (CI) illustrated that CT had unclear effects on inflammation (IL-6; CRP) and MVIC, but may have increased CK to a greater extent than PL and CJ conditions.

**Conclusion:**

There is no evidence of an additive effect of CJ and CWI when the treatments are used in conjunction with each other. On the contrary, combining CJ and CWI may result in slightly increased circulating CK.

## Introduction

Unaccustomed or strenuous exercise, such as marathon running, results in exercise-induced muscle damage (EIMD) (Belcastro et al., 1998) and inflammation (Starkie et al., 2001), which can last for up to a week. The process of EIMD is believed to occur in two stages, primary and secondary (Owens et al., 2019). The primary phase occurs during exercise due to metabolic stress from hypoxia of tissues (Tee et al., 2007), mechanical stress from repeated eccentric contractions (Owens et al., 2019), or both, as in marathon running. This initial damage triggers an inflammatory response (Owens et al., 2019) with damaged muscle fibers releasing pro-inflammatory cytokines that activate reactive oxygen species (ROS) generating enzymes and attracting neutrophils and macrophages to the tissue (Bowtell and Kelly, 2019). Following marathon running, research has demonstrated significant increases in interleukin-6 (IL-6) immediately after (Clifford et al., 2017), and C-reactive protein (CRP) after 24 h (Dimitriou et al., 2015; Bernat-Adell et al., 2021). Creatine kinase (CK) is typically used to assess EIMD and research demonstrates increases in CK 24 h after marathon (Bernat-Adell et al., 2021). As a result of the EIMD process, symptoms such as stiffness, swelling, delayed-onset muscle soreness (DOMS), and reduced muscle function can be present (Byrne and Eston, 2002; Tee et al., 2007). Detriments in muscle function can have deleterious effects on muscle performance, and marathon running has been shown to reduce maximum voluntary isometric contraction (MVIC) by 26.9% immediately after marathon, recovering to baseline levels by 48 h (Howatson et al., 2010). Therefore, recovery interventions to accelerate recovery and/or reduce damage are critical (Kellmann et al., 2018; Peake, 2019). Recovery interventions can take many forms, including cold water immersion (CWI) and cherry juice (CJ).

CWI is purported to limit inflammation, thereby mitigating any additional damage caused by the inflammatory response rather than exercise *per se* (Ihsan et al., 2014). CWI reduces muscle temperature, which alters the release of neurotransmitters that regulate fatigue, stimulates vasoconstriction, reduces tissue metabolism, and increases central blood volume (Leeder et al., 2012; Ihsan et al., 2014). CWI also increases hydrostatic pressure, causing fluid shifts and haemodilution which, when combined with increased central blood volume, assists metabolite efflux (Ihsan et al., 2016). These processes have the potential to ameliorate recovery by reducing inflammation, swelling, and soreness (Peake, 2019).

The evidence for CWI is somewhat inconsistent regarding the influence on inflammation, CK, and muscle function. Some authors report that CWI reduces CK (Leeder et al., 2012; Sanchez-Ureña et al., 2015; Dupuy et al., 2018), others report no effect (Bleakley et al., 2012; Hohenauer et al., 2015), and we have previously reported an increase in CK following CWI (Wilson et al., 2018). Furthermore, meta-analyses (Hohenauer et al., 2015; Dupuy et al., 2018) have reported that CWI has a small but non-significant effect on inflammation (IL-6, CRP) compared to a control. In terms of muscle function, several studies report that CWI does not ameliorate decrements in MVIC force (Eston and Peters, 1999; Sellwood et al., 2007; Goodall and Howatson, 2008; Jakeman et al., 2009), although some studies have reported positive effects (Bailey et al., 2007; Ascensão et al., 2011; Pournot et al., 2011). The lack of congruency in the literature for the efficacy of CWI is likely due to heterogeneous methodology. A range of different CWI protocols have been explored in the literature in terms of the exercise modality, duration, depth, temperature, and timing of the immersion following the exercise insult (Tipton et al., 2017). Inconsistent protocols combined with the individual variation of CK may therefore explain why the evidence surrounding the efficacy of CWI is equivocal. Notwithstanding, it is generally accepted that CWI is an effective recovery strategy for reducing soreness and limiting inflammation after exercise (Peake, 2019).

CJ is rich in phytochemicals which provide anti-inflammatory and antioxidant properties (Bongiovanni et al., 2020). Evidence consistently demonstrates that CJ effectively limits inflammation (IL-6, CRP) following both metabolic and mechanical exercise (Howatson et al., 2010; Bell et al., 2014b, 2015, 2016). Regarding muscle damage, current evidence has found no effect of CJ on CK (Howatson et al., 2010; Bell et al., 2016; Bowtell and Kelly, 2019). However, CK varies with exercise type and between individuals because of inherent high/low responders, training status, muscle fiber composition, and size (Brancaccio et al., 2007). Thus, the dynamic nature of CK coupled with the variety of populations in which CJ has been investigated may explain why no effect has been found. In terms of muscle function, several studies have reported significantly improved recovery of MVIC following CJ supplementation (Howatson et al., 2010; Bell et al., 2016; Quinlan and Hill, 2020; McHugh, 2022). Therefore, it is largely accepted that CJ is an effective recovery strategy (Peake, 2019).

Given that it is largely accepted that CJ and CWI are effective recovery strategies when utilized independently, Peake (2019) proposed that future research should investigate the effects of combining recovery strategies. The conjunction of CJ and CWI could be considered a “larger exposure” of recovery interventions, and therefore the concept of hormesis could be applied. As both CWI and CJ dampen hormesis (Peake et al., 2015), it is unknown whether the two combined work in synergy to limit inflammation and damage to a greater extent than either alone. Alternatively, when CWI and CJ are combined, the individual mechanisms of each may be altered and contradict each other, potentially causing detrimental effects on recovery. It is currently unknown whether combining recovery interventions such as CWI and CJ is excessive and has the potential to exacerbate damage in the same way.

While a physiological understanding of this novel concept is important, the practical rationale should also be taken into consideration. Investigations into typical recovery strategy usage in athletic populations have revealed that the majority (57%) of athletes use 1 or more (Crowther et al., 2017). Hence, it is clear that athletes are already using multiple recovery strategies without knowing the implications for recovery due to a lack of research in this area. As such, it is essential that research is carried out so that athletes and coaches know whether combining recovery interventions results in additive benefits or detrimental effects. Therefore, the aim of this study is to examine the efficacy of combining CWI and CJ (cocktail (CT)) compared to each in isolation, and a placebo, on muscle function (MVIC), markers of inflammation (IL-6, CRP), and muscle damage (CK) in trained endurance athletes following a trail marathon run.

## Methods

### Participants

A total of 39 healthy (non-smokers and no history of recent illness or disease) male endurance runners with an expected marathon completion time of ≤4.5 h participated in the study (Table 1). Five days before and during the study, participants were asked to avoid massage treatments, nutritional supplements (excluding the CJ and CT), non-steroidal anti-inflammatory drugs, and at least 2 days before, to avoid vigorous exercise. A one-way analysis of variance (ANOVA) was performed to check for significant differences between groups in participant completion times, personal records, and mass. No significant differences were identified.

**Table 1:** Participant physical characteristics, marathon personal records, and completion times.

	**Age (year)**	**Height (cm)**	**Mass (kg)**	**Marathon Personal Record (hh:mm:ss)**	**Marathon completion time (hh:mm:ss)**
PL	40.6 ± 7.2	174.7 ± 8.6	75.9 ± 10.2	03:20:27 ± 00:25:19	03:46:18 ± 00:34:04
CWI	41.3 ± 7.6	178.3 ± 7.6	79.2 ± 10.2	03:33:33 ± 00:27:10	03:43:05 ± 00:13:42
CJ	37.6 ± 7.8	176.0 ± 6.5	69.8 ± 6.9	03:21:00 ± 00:20:32	03:26:00 ± 00:23:44
CT	42.7 ± 4.7	178.2 ± 7.9	78.9 ± 10.7	03:26:00 ± 00:25:53	03:47:00 ± 00:23:30

Values are presented as mean ± SD.

### Design

Data was collected as part of a large-scale randomized placebo-controlled trial. Data relating to the PL and CWI conditions has been published previously as part of a different investigation (Wilson et al., 2018). Ethical clearance was approved by the institutional committee according to the Declaration of Helsinki. All participants completed a health questionnaire and consent form before being randomly assigned to either a PL (*n* = 10), CWI (*n* = 11), CJ (*n* = 10), or CT (*n* = 8) intervention group. Participants were familiar with all testing procedures before the marathon and baseline measures of all dependent variables were recorded. Participants then completed a competitive trail marathon run (nine laps of a 4.7 km outdoor loop) under their allocated intervention. During the marathon, participants were permitted to consume fluids, electrolytes, and food but not branched-chain amino acid supplements, protein supplements, antioxidants, or caffeine. Participants provided blood samples for analysis of all dependent variables immediately after marathon and at 24 h and 48 h after marathon.

### Dependent variables

Blood sampling was used to assess inflammation (IL-6; CRP) and muscle damage (CK). Approximately 8 ml of blood was collected from the antecubital vein into serum separation tubes immediately after marathon, 24 and 48 h after marathon. Specimens were centrifuged (3,000 rpm for 8 min), aliquoted and stored at −80°C for subsequent analysis, which was based upon known time-course responses (Kasapis and Thompson, 2005) (Table 2).

**Table 2:** List of blood markers measured at specific time points.

**Dependent variable**	**Baseline**	**Immediately post**	**24 h post**	**48 h post**
IL-6	✓	✓	✓	
CRP	✓		✓	✓
CK	✓		✓	✓

IL-6, interleukin-6; CRP, C-reactive protein; CK, creatine kinase.

#### IL-6

Plasma IL-6 concentration was determined using a quantitative sandwich (QS) enzyme-linked immunoassay (ELISA) technique (Quantikine, R&D Systems Europe Ltd., Abingdon, UK). The limit of quantification (LOQ), defined as the lowest concentration that could be distinguished from 0, was 0.38 pg/ml. The serum intra- and inter-assay precisions, determined by CV, were 3.8 and 8.3%, respectively.

#### CRP

Plasma CRP concentration was determined using a QS-ELISA (Quantikine, R&D Systems Europe Ltd., Abingdon, UK). The LOQ, defined as the lowest concentration that could be distinguished from 0, was 7.8 pg/ml with an intra- and inter-assay CV of 6.6 and 8.3%, respectively.

#### CK

Plasma CK-M concentrations were measured by simple step ELISA (ELISA, Abcam, Cambridge, UK). The reported assay ranges from 54.3 to 268.9 U/L, the minimum detection concentration (MDC) is 0.014 U/L, and the human serum intra- and inter-assay CVs are 3 and 9%, respectively.

#### MVIC

Maximal voluntary isometric contraction (MVIC) was measured on the self-reported dominant limb using an isokinetic dynamometer (Biodex 3, Biodex Medical Systems, Shirley, NY, USA). Following a standardized warm-up, MVIC was measured at a knee angle of 90° in accordance with previous studies (De Ruiter et al., 2004). Participants completed three maximal 5-s efforts. Peak values were used for analysis.

### Interventions

#### Placebo

A placebo was used instead of a control as blinding was not possible due to the nature of the interventions. The phytochemicals in CJ have been shown to aid recovery following a marathon (Bell et al., 2014a), therefore participants were informed that they would be consuming 30 ml of CJ twice a day (morning and evening) for 5 days before, the day of, and 2 days after the run. Instead, they received an isocaloric “fruit flavor” drink containing no phytochemicals. Following the completion of the marathon, participants were asked to rest for 10 min.

#### Cold water immersion

Immediately after the marathon, participants sat (wearing shorts, lower limbs and iliac crest fully immersed) in a mobile ice bath filled with water at 8°C (±0.5°) for 10 min. The ice bath was connected to a chiller unit (MiCool, iCool, Cranlea, UK) so that water temperature could be monitored and maintained within the desired parameters for the duration of the treatment.

#### Cherry juice

Participants consumed 30 ml of Montmorency CJ concentrate (CherryActive, Sunbury, UK) twice a day (morning and evening), for 5 days before, the day of, and 2 days after the run, in accordance with previous studies (Bell et al., 2014a). On the day of the run, participants took one bolus before and one bolus after marathon. Participants could have 30-ml CJ concentrated or diluted with water.

#### Cocktail

Participants received the CWI (10 min immersed in 8°C water after marathon) and CJ intervention (30 ml for 5 days before, the day of, and 2 days after the run) simultaneously.

### Data processing and analysis

To mitigate any confounding effects as a result of transient fluid shifts, plasma volume changes were calculated and biomarkers were adjusted accordingly using the equations by Dill and Costill (Dill and Costill, 1974). Raw data is reported as mean ± SD. Data were log-transformed for the blood markers to account for non-normal distribution, and all further data analysis is presented on back transformed data as mean ± 90% confidence intervals (CIs). No log transformation was required for the MVIC data. SPSS (IBM Corp., IBM SPSS Statistics for Windows, Version 25, Armonk, NY) was used for statistical analysis with a significance level of *P* < 0.05.

A mixed model ANOVA was used to analyse each dependent variable with a between-subjects factor of “intervention” (PL, CWI, CJ, and CT) and a within-subjects factor of “time” [baseline, immediately after (IL-6 only), 24 h, and 48 h after exercise) (Table 2). Mauchly's test of sphericity was used to assess the homogeneity of variance and, where necessary, Greenhouse-Geisser corrections were applied. Significant main effects or interaction effects were investigated using Bonferroni *post-hoc* pairwise comparisons. Partial eta squared (ηp^2^) was used to indicate the effect sizes for main effects and overall interaction with ≥0.01, ≥0.059, and ≥0.138 indicating small, moderate, and large effects, respectively (Cohen, 1988).

The smallest standardized worthwhile (Cohen) change (SWC) was calculated as 0.2 times the between-subjects standard deviation (Batterham and Hopkins, 2006) for baseline values (back-transformed) of all participants. A change greater than the SWC indicates a practically meaningful response (Hopkins, 2004; Swinton et al., 2018). Cohen's *d* effect sizes were used to determine the magnitude of the difference between groups with <0.2, indicating a trivial effect, 0.2 a small effect, 0.5 a medium effect, 0.8 a large effect, and >1.2 a very large effect (Cohen, 1988).

## Results

The average (mean ± SD) IL-6, CRP, CK, and MVIC values for each group at each time point are shown in Table 3.

**Table 3:** Group averages for all variables at each time point.

		**PL**	**CWI**	**CJ**	**CT**
IL-6 (pg/ml)	Baseline	42.6 ± 104.3	75.9 ± 157.3	29.7 ± 49.3	21.7 ± 55.2
	Post	56.6 ± 115.8	89.5 ± 151.1	73.6 ± 89.3	34.2 ± 63.1
	24 h	47.0 ± 110.4	82.8 ± 169.9	27.6 ± 37.6	32.7 ± 71.7
CRP (ng ML^−1^)	Baseline	1,625.5 ± 3,837.9	586.1 ± 378.0	951.5 ± 691.0	474.3 ± 563.5
	24 h	8,439.3 ± 7,002.5	4,890.5 ± 3,615.0	6,403.0 ± 3,221.0	6,905.6 ± 2,669.4
	48 h	7,012.7 ± 8,766.4	3,449.8 ± 2,552.7	4,364.0 ± 2,985.2	2,866.7 ± 1,318.9
CK (U L^−1^)	Baseline	31.2 ± 18.0	25.0 ± 14.5	22.0 ± 11.6	30.4 ± 9.6
	24 h	50.3 ± 29.0	82.9 ± 100.8	51.8 ± 50.2	126.9 ± 78.5
	48 h	51.4 ± 41.8	39.2 ± 23.1	26.6 ± 14.6	61.5 ± 28.0
MVIC (N)	Baseline	208.8 ± 34.7	211.7 ± 40.6	213.9 ± 52.7	211.6 ± 48.5
	Post	179.8 ± 31.7	191.4 ± 38.7	187.8 ± 51.3	171.0 ± 46.2
	24 h	203.5 ± 33.5	207.4 ± 33.8	211.2 ± 57.1	200.9 ± 42.7
	48 h	210.5 ± 30.7	215.9 ± 38.6	224.1 ± 51.8	234.7 ± 55.3

Data are presented as mean ± standard deviation of the absolute raw values. PL, placebo; CWI, cold water immersion; CJ, cherry juice; CT, cocktail.

### IL-6

**Figure 1:**
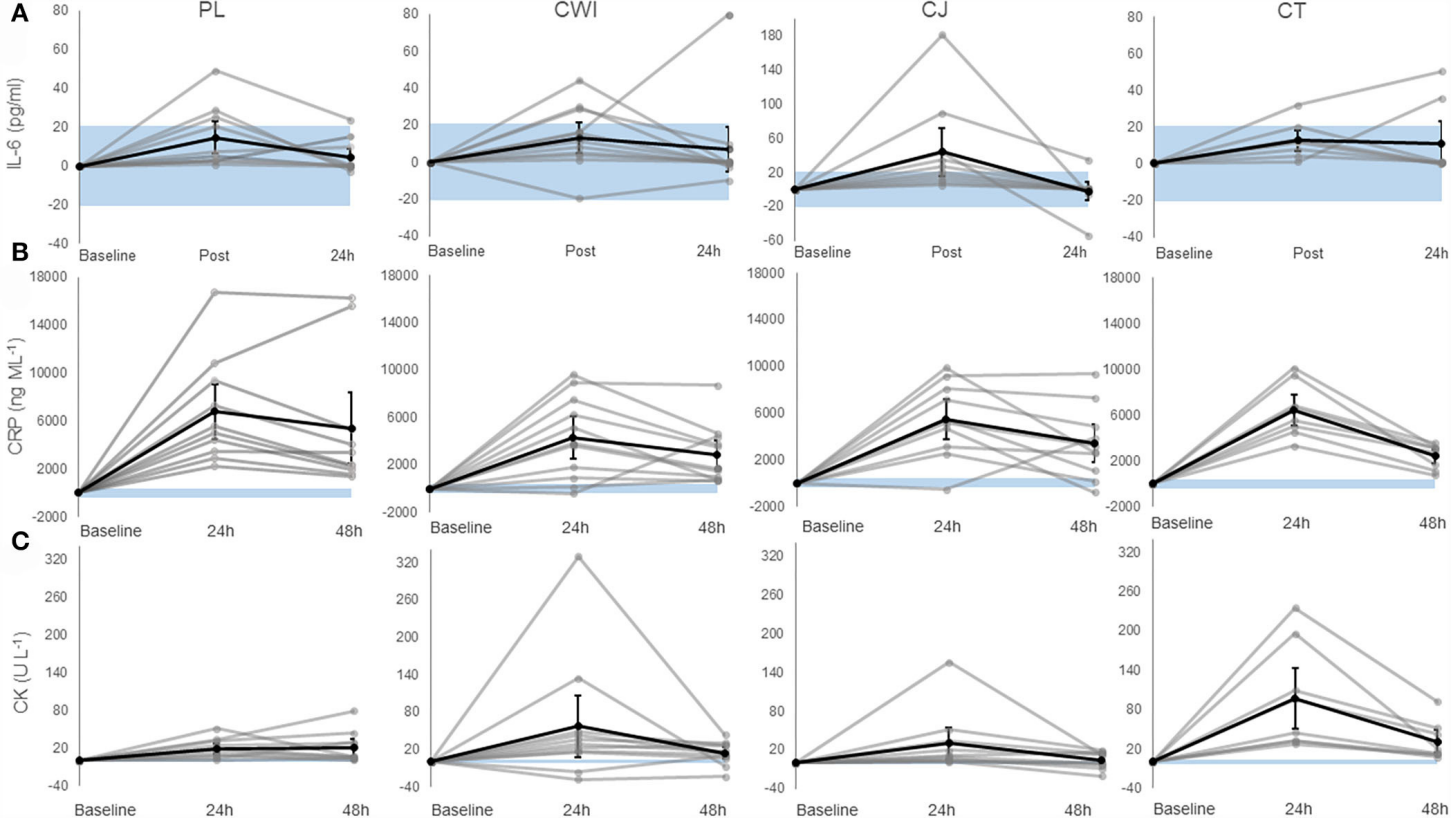
**(A)** Change in IL-6 in the placebo, cold water immersion, cherry juice, and cocktail group (from left to right). **(B)** Change in CRP in the placebo, cold water immersion, cherry juice, and cocktail group (from left to right). **(C)** Change in CK in the placebo, cold water immersion, cherry juice, and cocktail group (from left to right). Blue shaded area represents smallest worthwhile change (±20.7, ±399.1, ±2.8 for IL-6, CRP, and CK, respectively); black line represents the corresponding group average across time and 90% confidence limits; gray lines represent individual responses across time. Note: Value shown at post is the change from baseline to post and value shown at 24 h post is the change from baseline to 24 h post and so forth. Altered axis scale was used for IL-6 in CJ group.

There was a significant main effect of time [*F*_(1.407, 49.240)_ = 37.054, *P* = 0.000, ηp^2^ = 0.514] and *post-hoc* pairwise comparisons confirmed the differences were significant between baseline-immediately and 24 h after marathon. Although there was a significant increase in IL-6 across time, only the CJ group had an average change over time greater than the SWC (Figure 1A). There was no significant difference between groups [*F*_(3, 35)_ = 0.217, *P* = 0.884, ηp^2^ = 0.018] and no significant group × time interaction effect [*F*_(4.221, 49.240)_ = 0.645, *P* = 0.641, ηp^2^ = 0.052]. Group comparisons were largely inconclusive between the CT group and all other intervention groups immediately and 24 h after as almost all CIs crossed 0 (Table 4). However, the 90% CI for the CJ and CT comparison from baseline to immediately after did not cross 0 with a large effect size of 0.8, with a smaller increase in CT compared to CJ (Table 4).

**Table 4:** Difference between groups and group comparisons for each dependent variable.

		**Difference in means**^**a**^ ±**CL**^**b**^			**Effect sizes**^**a**^ **(lower ES – upper ES)**^**bc**^		
		**CT/PL**	**CT/CWI**	**CT/CJ**	**CT/PL**	**CT/CWI**	**CT/CJ**
IL-6 (pg/ml)	B-Post	1.9 ± 10.7	0.4 ± 10.8	31.2 ± 30.6	0.1 (−0.7, −1.0)	0.0 (−0.8, −0.8)	0.8 (−0.1, −1.6)
	B-24 h	−6.5 ± 13.3	−4.2 ± 17.9	−13.3 ± 17.1	−0.4 (−1.3, −0.4)	−0.2 (−1.0, −0.6)	−0.6 (−1.5, −0.2)
CRP (ng ML^−1^)	B-24 h	338.2 ± 2,860.6	−2,137.2 ± 2,349.9	−976.0 ± 2,311.4	0.1 (−0.7, −0.9)	−0.7 (−1.5, −0.1)	−0.3 (−1.2, −0.5)
	B-48 h	3,001.4 ± 3,204.6	456.7 ± 1,459.8	1,008.9 ± 1,851.7	0.7 (−0.2, −1.5)	0.2 (−0.6, −1.0)	0.4 (−0.4, −1.2)
CK (U L^−1^)	B-24 h	−77.4 ± 50.1	−39.2 ± 71.8	−66.8 ± 55.8	−1.4 (−2.4, −0.5)	−0.4 (−1.2, −0.4)	−1.1 (−1.9, −0.2)
	B-48 h	−10.7 ± 22.8	−7.2 ± 20.5	−26.5 ± 19.5	−0.4 (−1.2, −0.4)	−0.7 (−1.6, −0.1)	−1.2 (−2.1, −0.3)
MVIC (N)	B-Post	11.7 ± 24.7	20.3 ± 24.8	14.6 ± 27.4	0.4 (−0.4, −1.2)	0.7 (−0.1, −1.5)	0.5 (−0.4, −1.3)
	B-24 h	5.4 ± 17.4	6.4 ± 15.9	8.0 ± 19.9	0.3 (−0.6, −1.1)	0.3 (−0.5, −1.1)	0.3 (−0.5, −1.2)
	B-48 h	−21.4 ± 25.9	−18.8 ± 25.3	−12.8 ± 24.3	−0.7 (−1.6, −0.1)	−0.6 (−1.5, −0.2)	−0.5 (−1.3, −0.4)

ES, Cohen's d effect size; CT, Cocktail group; PL, Placebo group; CWI, Cold-water immersion group; CJ, Cherry juice group. Data are reported on the back transformed data as the difference in means of the absolute changes from baseline to immediately/24 h/48 h post depending on the time course of the variable.

^a^Differences in means and effects sizes calculated as the second named group (PL/CWI/CJ) minus the first named group (CT); a negative value indicates that the CT group had a larger change compared to the other group.

^b^Add and subtract the 90% confidence limits to obtain the upper and lower limits.

^c^Critical t-value for 90% distribution used for upper and lower effects.

### CRP

There was a significant main effect of time [*F*_(1.599, 55.960)_ = 97.271, *P* = 0.000, ηp^2^ = 0.735] and *post-hoc* pairwise comparisons confirmed the difference was significant between baseline-24 h after, 24 h after-48 h after, and baseline-48 h after. All groups had an average change over time greater than the SWC (Figure 1B). There was no significant difference between groups [*F*_(3, 35)_ = 0.348, *P* = 0.791, ηp^2^ = 0.029] and no significant interaction effect [*F*_(4.797, 55.960)_ = 2.166, *P* = 0.073, ηp^2^ = 0.157]. Group comparisons based on mean differences and effect sizes between the CT group and all other intervention groups were inconclusive 24 h and 48 h after as all CIs crossed 0 (Table 4).

### CK

There was a significant main effect of time [*F*_(1.616, 56.545)_ = 29.935, *P* = 0.000, ηp^2^ = 0.461] and *post-hoc* pairwise comparisons confirmed the difference was significant between baseline-24 h after, 24 h after-48 h after, and baseline-48 h after. All groups had an average change over time greater than the SWC (Figure 1C). There was no significant difference between groups [*F*_(3, 35)_ = 2.815, *P* = 0.053, ηp^2^ = 0.194] and no significant interaction effect [*F*_(4.847, 56.545)_ = 1.587, *P* = 0.180, ηp^2^ = 0.120]. Group comparisons revealed that the increase in CK over time was greater in the CT group compared to the CJ group at 24 h and 48 h after marathon as the CIs did not cross 0 with large and very large effect sizes of 1.1 and 1.2, respectively (Table 4). Similarly, the change in CK across time was greater in the CT group compared to the placebo 24 h after marathon as the 90% CI did not cross 0 with a very large effect size of 1.4 (Table 4). However, the differences in CK over time between the CT and CWI group at 24 h and 48 h after marathon and the placebo group at 48 h after marathon were inconclusive as the CIs crossed 0 (Table 4).

### MVIC

**Figure 2:**
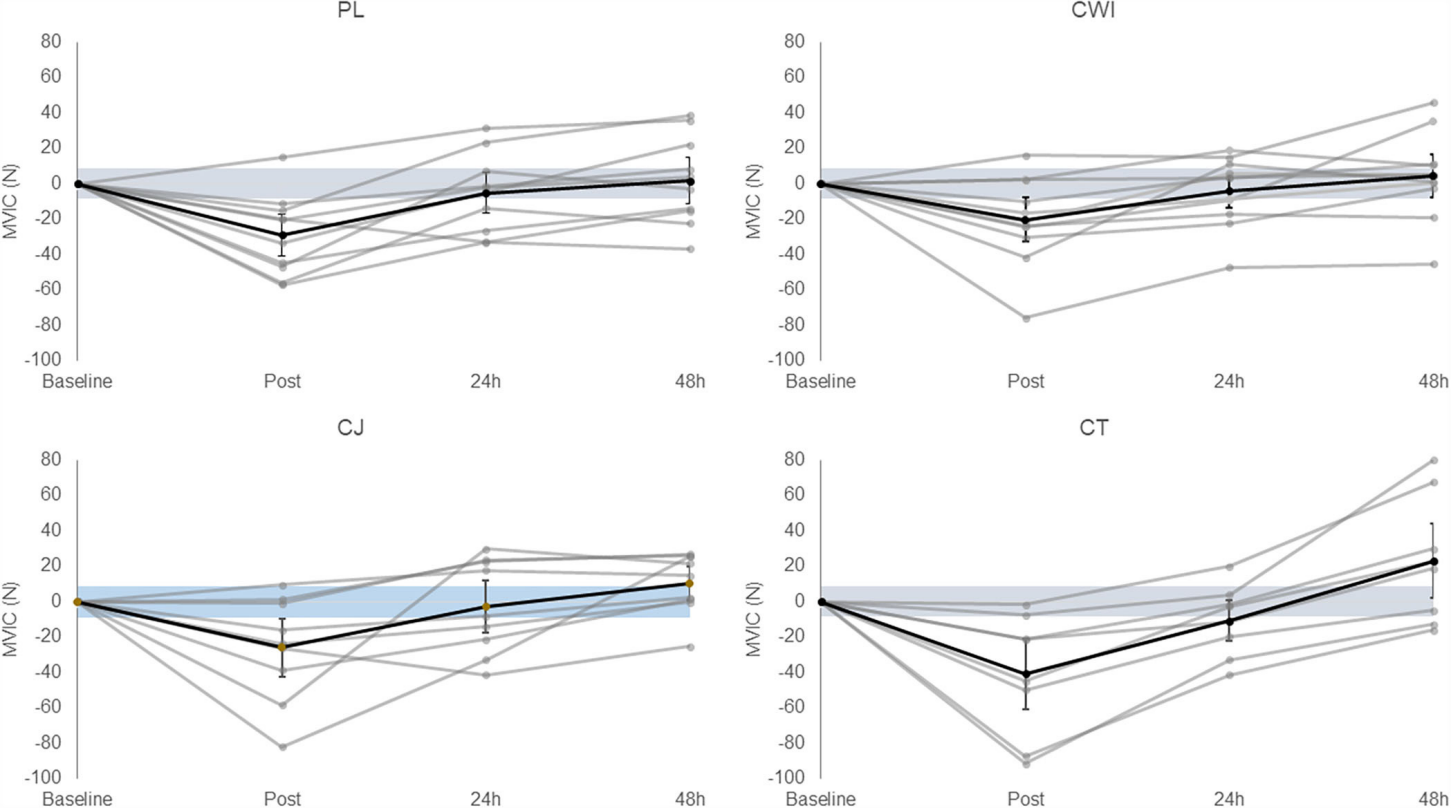
Change in MVIC in the placebo, cold water immersion, cherry juice, and cocktail group (from left to right, top to bottom). Blue shaded area represents smallest worthwhile change (±8.46); black line represents the corresponding group average across time and 90% confidence limits; gray lines represent individual responses across time. Value shown at post is the change from baseline to post and value shown at 24 h post is the change from baseline to 24 h post and so forth.

There was a significant main effect of time [*F*_(3, 129)_ = 34.382, *P* = 0.000, ηp^2^ = 0.444] and *post-hoc* pairwise comparisons confirmed the difference was significant between baseline-post, post-24 h post, post-48 h post, and 24 h post-48 h post. All groups had an average change over time greater than the SWC (Figure 2). There was no significant difference between groups [*F*_(4, 43)_ = 0.053, *P* = 0.995, ηp^2^ = 0.005]. Group comparisons were largely inconclusive between the cocktail group and all other intervention groups immediately, 24 h, and 48 h after as all CIs crossed 0 (Table 4).

## Discussion

This study examined the efficacy of combining CWI and CJ (CT) compared to each intervention in isolation, and a placebo on markers of inflammation (IL-6, CRP), muscle damage (CK), and muscle function (MVIC) in trained endurance athletes following a marathon run. The marathon run led to increases in markers of inflammation and muscle damage, and reductions in muscle function. In terms of comparisons between the interventions, there was limited evidence to suggest that the CT intervention attenuated inflammation or strength decrements to a greater extent than the placebo, CWI or CJ intervention based on IL-6, CRP, and MVIC as the CIs were largely inconclusive. Evidence based on CIs seemed to demonstrate that the CT increased CK to a greater extent than the PL 24 h after marathon and the CJ group at 24 h and 48 h after marathon, but not CWI. Thus, the data were largely inconclusive and overall, there is no clear beneficial or harmful effect of CT on inflammation, CK, or MVIC following a marathon run.

The inconclusive evidence surrounding the effects of CT on inflammation could indicate a conflict in the physiological mechanisms of CWI and CJ. For example, CWI reduces tissue temperature and as a corollary of this, blood flow is reduced *via* vasoconstriction (Ihsan et al., 2016; Peake, 2019). However, the phytochemicals (anthocyanins, hydroxycinnamates, and Flavan-3-ols), which provide CJ with its anti-inflammatory properties, are delivered *via* the blood (Ferretti et al., 2010). Such properties include blocking the NF-kB pathway, inhibiting cyclooxygenase-2 and upregulating antioxidant enzymes (Ferretti et al., 2010; Bowtell and Kelly, 2019). Therefore, vasoconstriction by CWI may antagonize these actions by limiting delivery of the bioactive compounds. Moreover, CWI reduces metabolic rate (Ihsan et al., 2016) and therefore may counteract the upregulation of antioxidant enzymes by CJ (Bowtell and Kelly, 2019). This reduced enzyme activity compared to CJ alone may be amplified by the low temperatures of CWI as enzymes are subject to cold denaturation (Tipton et al., 2017). Thus, one of these physiological phenomena could explain the inconclusive effects of the CT on inflammation. As the results are unclear, it is difficult to substantiate that the two definitely contradict, therefore a synergistic perspective is considered later in the discussion regarding muscle damage.

From a methodological standpoint, there is consistent evidence showing CWI improves perceptual recovery (Peake, 2017); however, the evidence surrounding its effect on inflammation is equivocal (Tipton et al., 2017). Peake (2017) demonstrated that CWI was no more effective than active recovery in attenuating inflammation. Dupuy et al. (2018) corroborated this, reporting that CWI has no significant effects on IL-6 and CRP. Given there is consistent evidence surrounding the effect of CJ on inflammation (Peake, 2019), the unclear effects of the CT may be attributable to the CWI; the CIs demonstrated that the CT was more inflammatory than the CJ and PL interventions, neither of which employed CWI. This also suggests that the CWI aspect of the CT was responsible. This study used a protocol of 8°C for 10 min, which is a temperature (Mawhinney et al., 2020) deems noxious. Paradoxically, Mawhinney et al. (2020) showed that CWI at 8°C led to an increase in deep muscle perfusion compared to non-noxious temperatures. Therefore, noxious cooling may have the capacity to accentuate the inflammatory response, subsequently increasing membrane permeability and efflux of CK. This may elucidate the muscle damage findings; however, this can only be inferred. As such, it is unclear whether the CK findings can be attributed to the CWI protocol or the combination of CWI and CJ.

In the event that the combination of the two treatments was causing more damage and not solely the CWI, it may be because both CWI and CJ work on the premise of dampening hormesis (Peake et al., 2015). This combination may excessively decrease inflammatory signaling, preventing tissue recovery, and prolonging local damage. For instance, CWI reduces blood flow, tissue metabolism, and the capacity to produce growth factors and chemotaxic factors (Peake et al., 2015). These factors are required to stimulate neutrophil/macrophage infiltration and satellite cell proliferation and differentiation (Peake et al., 2015; Ihsan et al., 2016). In isolation, this reduced inflammatory signaling may protect healthy bystander cells not damaged by the initial insult. However, CJ also reduces inflammation by blocking the NF-kB pathway, inhibiting cyclooxygenase-2, and upregulating antioxidant enzymes (Bowtell and Kelly, 2019). This reduces the production of ROS and pro-inflammatory cytokines (IL-6; TNFα) (Beconcini et al., 2020). Given the largely inconclusive effects of CT on inflammation, it cannot be ruled out that, theoretically, CT could excessively dampen the inflammatory response, thereby preventing remodeling and potentially augmenting damage (Isaacs et al., 2019).

Despite this, it is important to reiterate that the findings of this study are not conclusive enough to label the CT intervention harmful. Moreover, there is a research showing that raw CK values should not be used as a reflection of the level of muscle damage (Baird et al., 2012). This is because CK can be influenced by gender, age, ethnicity, training status, exercise modality, and genetics (Brancaccio et al., 2007) making it highly variable. Thus, the vast inconsistency in the CK response means that, in isolation, it is not an accurate representation of muscle damage.

There was no clear evidence showing CT had a beneficial or harmful effect on inflammation compared to the PL. However, the CIs seemed to demonstrate that CT was more damaging (CK) than PL 24 h after marathon. Placebos can facilitate participant recruitment and retention, eliminate bias (Hróbjartsson et al., 2011) and enable the effectiveness of a treatment to be analyzed (Castro, 2007). However, the placebo response is markedly different between individuals (Bérdi et al., 2011) and seems to be closely linked to expectancy of effects and treatment belief (McClung and Collins, 2007). For example, if an individual expects the treatment to be effective, it may lead to a placebo effect whereby changes are seen despite the intervention being “inert” (McClung and Collins, 2007). Therefore, this could have influenced the results as evidence has shown that the placebo effect can manipulate and dampen inflammation through the endocrine system (Hunter, 2007). Hence, a placebo effect may have influenced the results in the CT condition.

Moreover, this study addressed only the acute effects of combining CJ and CWI. Although there is no research investigating long-term CJ supplementation, chronic supplementation of vitamin E and C, which are akin to CJ, and regular use of CWI have been investigated. Long-term use of CWI and vitamin C/E can blunt anabolic signaling pathways and impair resistance training adaptations (Broatch et al., 2018; Higgins et al., 2020; Malta et al., 2021) though no effect on long-term endurance training adaptation or performance has been reported for either (Broatch et al., 2018; Mason et al., 2020; Malta et al., 2021). Therefore, the combination of CWI and CJ over time may warrant research into the effects on long-term training adaptation and performance.

With regards to muscle function, although there was a significant reduction in MVIC immediately following the marathon, and recovery to baseline levels by 48 h post-exercise, there were no differences between groups at any time-point. These findings are in contrast to previous research, which demonstrated improved MVIC recovery in the CJ group compared to the placebo after a marathon (Howatson et al., 2010). Marathon completion times for the CJ groups are comparable between studies (~3.5 h), but the participants in the placebo group completed the marathon significantly faster in the present study than those in the Howatson et al.'s study (Howatson et al., 2010) (03:46:18 ± 00:34:04 vs. 4:15:48 ± 1:01:22 h:min:s, respectively). This may account in part for the differing results presented here. The finding that CWI did not attenuate decrements in muscle function is in line with a number of previous studies (Eston and Peters, 1999; Sellwood et al., 2007; Goodall and Howatson, 2008; Jakeman et al., 2009). Given that neither CWI nor CJ in isolation positively influenced MVIC outcomes post-exercise, it is not surprising that there was no beneficial effect of CT on muscle function in this study.

The limitations of this study should be addressed. The recovery effects of CWI vary with body composition and body fat levels (Stephens et al., 2018). However, this was not measured, only total body mass was reported. Therefore, this may have impacted the findings of this study, and future studies should use a CWI protocol, which accounts for individual body composition. It is unknown whether the effects of CJ vary in the same way. Thus far, no dose-dependent relationship has been reported (Gao and Chilibeck, 2020; Wangdi et al., 2021) yet doses in the literature vary from the equivalent of 45–270 cherries/day (Kelley et al., 2018). Therefore, optimal CJ benefits could require a dosage per kilogram of body mass in the same way daily protein requirements do. As already alluded to, the treatment temperature utilized for CWI was likely lower than optimal, and this may have influenced the results for both CWI and CT. Additionally, this study used small sample sizes, which increases the chance of error and reduces statistical power (Sainani, 2009). Therefore, future studies should aim to recruit larger samples.

## Conclusion

In terms of comparisons between interventions, CT demonstrated an unclear effect on inflammation (IL-6; CRP) and muscle function (MVIC) compared to the CWI, CJ, and PL interventions. The CIs insinuated that CT increased circulating CK to a greater extent than the PL and CJ group. These novel findings provide evidence for coaches and athletes that combining CWI and CJ is neither beneficial nor harmful. The effects of CT compared to PL were unclear, which suggests a placebo effect may have influenced the results. Therefore, future studies should continue to ensure effective placebo interventions are implemented or a measure of treatment belief is included. These novel findings indicate that combining CWI and CJ was neither beneficial nor harmful for recovery following a trail marathon. As such, athletes and coaches may want to avoid utilizing a cocktail (CWI and CJ) approach until conclusive evidence of recovery or performance benefits is presented. Future studies should investigate the recovery effects of combining CJ and CWI following different exercise modalities to facilitate a better understanding of the mechanisms. Furthermore, inflammation does not necessarily need to be limited to prevent decrements in muscle function and subsequent performance. Therefore, further investigations should examine additional muscle function and subjective outcomes to assess the practical impact on athletic performance.

## Data availability statement

The raw data supporting the conclusions of this article will be made available by the authors, without undue reservation.

## Ethics statement

The studies involving human participants were reviewed and approved by London Sport Institute Research Ethics sub-committee, Middlesex University. The patients/participants provided their written informed consent to participate in this study.

## Author contributions

LW, EC, and LD: study conception and design. LW, EC, LD, KP, SS, TF, and AW: data collection. LW, EC, LD, KP, SS, TF, FH, MG, AW, and ID: analysis and interpretation of results. ID, EC, and LW: draft manuscript preparation. All authors reviewed the results and approved the final version of the manuscript.

## Conflict of interest

The authors declare that the research was conducted in the absence of any commercial or financial relationships that could be construed as a potential conflict of interest.

## Publisher's note

All claims expressed in this article are solely those of the authors and do not necessarily represent those of their affiliated organizations, or those of the publisher, the editors and the reviewers. Any product that may be evaluated in this article, or claim that may be made by its manufacturer, is not guaranteed or endorsed by the publisher.
